# Classification of paroxysmal atrial fibrillation using sinus rhythm electrocardiograms using the symmetric projection attractor reconstruction method

**DOI:** 10.1038/s41598-026-37491-1

**Published:** 2026-02-18

**Authors:** Steven Creasy, Gregory Y. H. Lip, Gary Tse, Manasi Nandi, Kamalan Jeevaratnam, Philip J. Aston

**Affiliations:** 1 Department of Comparative Biomedical Sciences, School of Veterinary Medicine, University of Surrey, Daphne Jackson Road, Guildford, GU2 7AL UK; 2 School of Mathematics and Physics, University of Surrey, Guildford, UK; 3 Liverpool Centre for Cardiovascular Science, University of Liverpool, Liverpool John Moores University and Liverpool Heart and Chest Hospital, Liverpool, UK; 4 Danish Center for Health Services Research, Department of Clinical Medicine, Aalborg University, Aalborg, Denmark; 5 Cardiovascular Analytics Group, PowerHealth Limited, Kwai Chung, Hong Kong China; 6 Tianjin Key Laboratory of Ionic-Molecular Function of Cardiovascular Disease, Department of Cardiology, Tianjin Institute of Cardiology, Second Hospital of Tianjin Medical University, Tianjin, China; 7 School of Nursing and Health Studies, Hong Kong Metropolitan University, Kowloon, Hong Kong China; 8 School of Cancer and Pharmaceutical Sciences, Faculty of Life Sciences and Medicine, King’s College London, London, UK

**Keywords:** Paroxysmal atrial fibrillation, Machine learning, Prediction, Electrocardiogram, SPAR analysis, Cardiology, Computational biology and bioinformatics, Diseases, Medical research

## Abstract

Atrial fibrillation is the most commonly encountered cardiac arrhythmia, increasing stroke risk and mortality. Paroxysmal atrial fibrillation (PAF) can be challenging to detect because arrhythmias occur intermittently. We have been able to classify PAF patients from sinus rhythm electrocardiograms (ECG), using a signal processing technique, Symmetric Projector Attractor Reconstruction, which transforms the ECG time-series into a quantifiable two-dimensional image termed an attractor. To optimise this methodology, we investigated the impact of varying parameters within the SPAR method, choice of lead, ECG sampling frequency and machine learning model choice. We determined that using a K nearest neighbours (KNN) model with 125 Hz ECG sampling frequency, using specific features that quantified the density of the attractor, gave a classification accuracy of 81.2%. When using a decision tree model it was found that the sensitivity was 72.5% which shows an obvious improvement over 30-day long term monitoring which has a sensitivity of 34%. The results of this paper present consideration for the application of a new method to the clinically relevant problem of aiding detection of PAF and conjectures as to the reasoning behind these results. Further investigation in larger cohorts is needed to fully elucidate these findings.

## Introduction

Atrial fibrillation (AF) is a common and under-diagnosed heart condition associated with increased risk of stroke, heart failure, dementia, and mortality^1,2^. AF patients have a stroke risk three to five times greater than healthy patients^3^ and studies have shown the 5-year mortality rate is around 50% for both men and women^4^. It is estimated to affect at least 3.4% of the adult population^5^.

Currently, diagnosis of clinical AF requires witnessing an episode of at least 30 s according to the European Society of Cardiology guidelines which defines AF in the following way: “A supraventricular tachyarrhythmia with uncoordinated atrial electrical activation and consequently ineffective atrial contraction”^6^. Electrographic characteristics for the diagnosis of AF include: irregularly irregular R-R intervals, absence of distinct repeating P waves, and irregular atrial activations^6^. A recent study in the middle east with data collected from hospitals in Jordan showed that around one third of AF cases presented as paroxysmal atrial fibrillation (PAF)^7^, which are recurrent episodes of AF alternating with sinus rhythm that terminate spontaneously or with intervention within 7 days^6^.

Screening for PAF is challenging due to the low diagnostic yield of a single electrocardiogram (ECG). Most recordings have a low AF burden (the percentage time of a recording that a patient is in AF) and long-term ECG monitoring is cumbersome and uncomfortable^8^. As there is likely to be an increase in PAF prevalence due to the ageing population^9,10^, more research to identify potential screening methods for AF is in progress^11^. Previous studies and reviews have investigated the accuracy of different types of screening. It has been shown that systematic screening (being invited for regular ECGs) and opportunistic screening (ECGs taken when heart palpitations occur) both show an increased ability to detect AF^12,13^ over routine practice, which is defined as “diagnoses made during routine care, incidentally or following presentation with indications for AF testing, that were subsequently confirmed by 12-lead or continuous ambulatory ECG interpreted by a GP, a specialist or a suitably trained ECG technician or nurse“^13^.

Previous analysis on the diagnosis of AF can be split into two categories namely those with the intention of detecting a patient in AF, and those aiming to diagnose a patient currently in sinus rhythm outside of an AF episode^14,15^. For detection of PAF, due to the potential for episodes to be as short as 30 s, the use of artificial intelligence (AI) has aided with diagnosis from sinus rhythm ECGs. AI algorithms can recognise changes to an ECG that may not be obvious to a cardiologist using conventional ECG analysis. AI models also have the advantage of being able to be trained from many thousands of records allowing for direct and rapid comparison to other known PAF cases, and can be provided with non-conventional features from the ECG.

In 2019 a study employed AI methods to classify patients who had evidence of at least one episode of rhythm of atrial fibrillation or atrial flutter, from their sinus rhythm ECG signals (500 Hz) acquired 31 days preceding the AF event^15^. 649,931 10-second 12-lead ECGs from 180, 922 adult patients were given to a convolutional neural network (CNN), and the model was able to achieve an accuracy of 79.4% with the sensitivity of 79.0% and specificity of 79.5%. This meant that the model was able to predict both cases (AF) and controls (healthy) with equal ability. It is probable that the success of this model was determined by its relatively high sample size and the availability of 12 leads, as CNN models are data hungry. The generalisability of this model to other datasets is yet to be determined and will be important to support clinical deployment.

Symmetric Projector Attractor Reconstruction (SPAR)^16,17^ is a relatively new signal processing technique that has previously been used to retrospectively classify ECG signals for a range of conditions. SPAR transforms any cyclic time-series data, such as ECG or blood pressure signals, into a corresponding two-dimensional image. SPAR amplifies small morphology or variability changes in the underlying signal and does not rely on the accurate detection of specific features of interest, such as P, Q, R, S, T points, but rather appraises the waveform as a whole.

SPAR has been used to support classification of groups from ECG signals including sex differences in humans from 12 lead ECGs^18^, a mouse model of Brugada-syndrome from lead I and lead II ECGs^19^, and PAF in horses from a single lead ECG^20^.

In this study we applied the SPAR method using short sinus rhythm ECGs taken from patients with a known AF episode, to establish whether it could successfully classify patients with and without evidence of an AF episode from an otherwise clinically unremarkable signal. In particular the sinus rhythm ECG was taken after the known AF episode, presenting a different approach to previous literature in this area. This was chosen to determine the viability of using a machine learning model to detect AF in patients entering primary care having experienced AF symptoms. This relieves the need for subsequent AF episodes to occur as in current primary care practice.

We hypothesised that the sampling frequency of the ECG recordings, along with choice of lead, AI model, and the parameters of the SPAR method (described in Sect. 3.3) would impact the overall accuracy and sought to find the optimal configuration of all of these parameters for the detection of PAF.

## Results

**Fig. 1:**
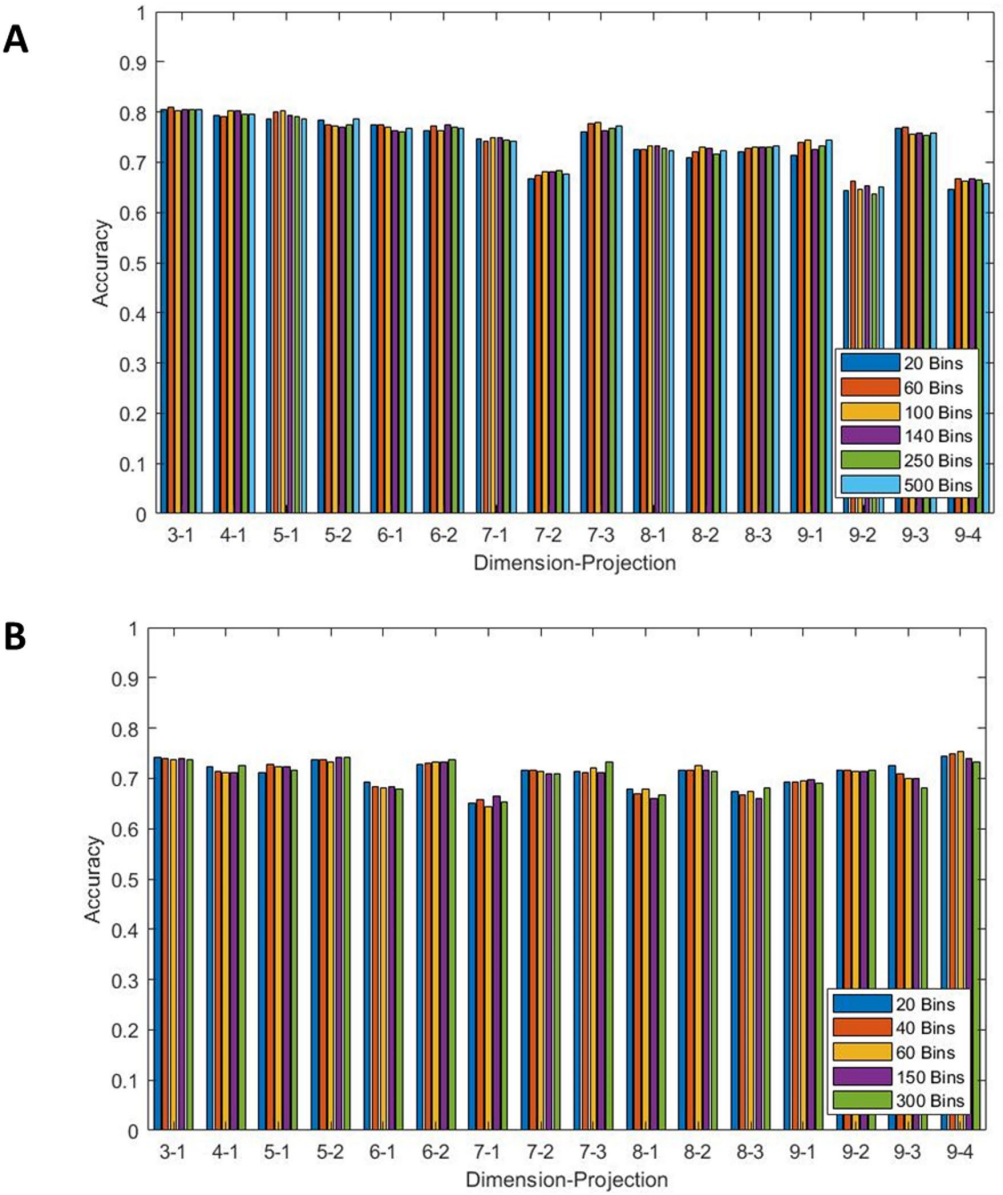
For both panels signals sampled at 500 Hz were used together with a KNN model for all projections of dimensions 3–9. The maximum accuracy across all 12 leads is shown for (**A**) the θ density features and (**B**) the r density features for a range of bin values.

Figure 1A and B show the accuracy obtained using signals sampled at 500 Hz sampling frequency and a KNN model for a range of resolutions for θ and r densities respectively with the maximum value taken across all 12 leads for each N and k. Results for all resolution values stated in Sect. 3.4 were obtained; however, for clarity of the figure, some results were omitted. The most frequent leads for maximal results varied between feature sets but remained consistent across all resolutions with r density features showing highest accuracies for leads V3, V4 and V6, whereas θ density features achieved best results for leads I, aVL and V6. Lead V1 is often considered the most sensitive for detecting AF due to its proximity to the right atrium^21^ and so at first inspection this may be a curious result. However, we are not trying to detect an occurrence of AF but a susceptibility of the patient to AF. The SPAR method can be applied to any approximately periodic signal and as such can be considered to be lead agnostic. Similarly, machine learning techniques look for patterns not obvious to human observers and as such may not be detecting classical rhythm changes. This is why as part of this study we considered all leads individually to avoid any limitation on the results due to data selection. As leads I and II are most common for handheld devices this could help explain the poor accuracy when using such devices for detection. Similar results were generated for decision tree models which performed similarly to KNN models for r density features. However, the results for θ density features showed a consistent decrease in accuracy of around 8%. Based on the trend of these results, and particularly those with the highest accuracies, it was decided that for a KNN model 20 bins would be used for the r density features and 60 bins would be used for the θ density features. Similarly for a decision tree model, 40 bins was chosen for r density features and 100 bins for θ density features. These were chosen due to their higher accuracies across the values of N and k. Specific focus was on *N* = 3 k = 1, and *N* = 5 k = 1 for θ density features and *N* = 3 k = 1, and *N* = 9 k = 4 for r density features due to their higher accuracies compared to other N and k combinations.

**Fig. 2:**
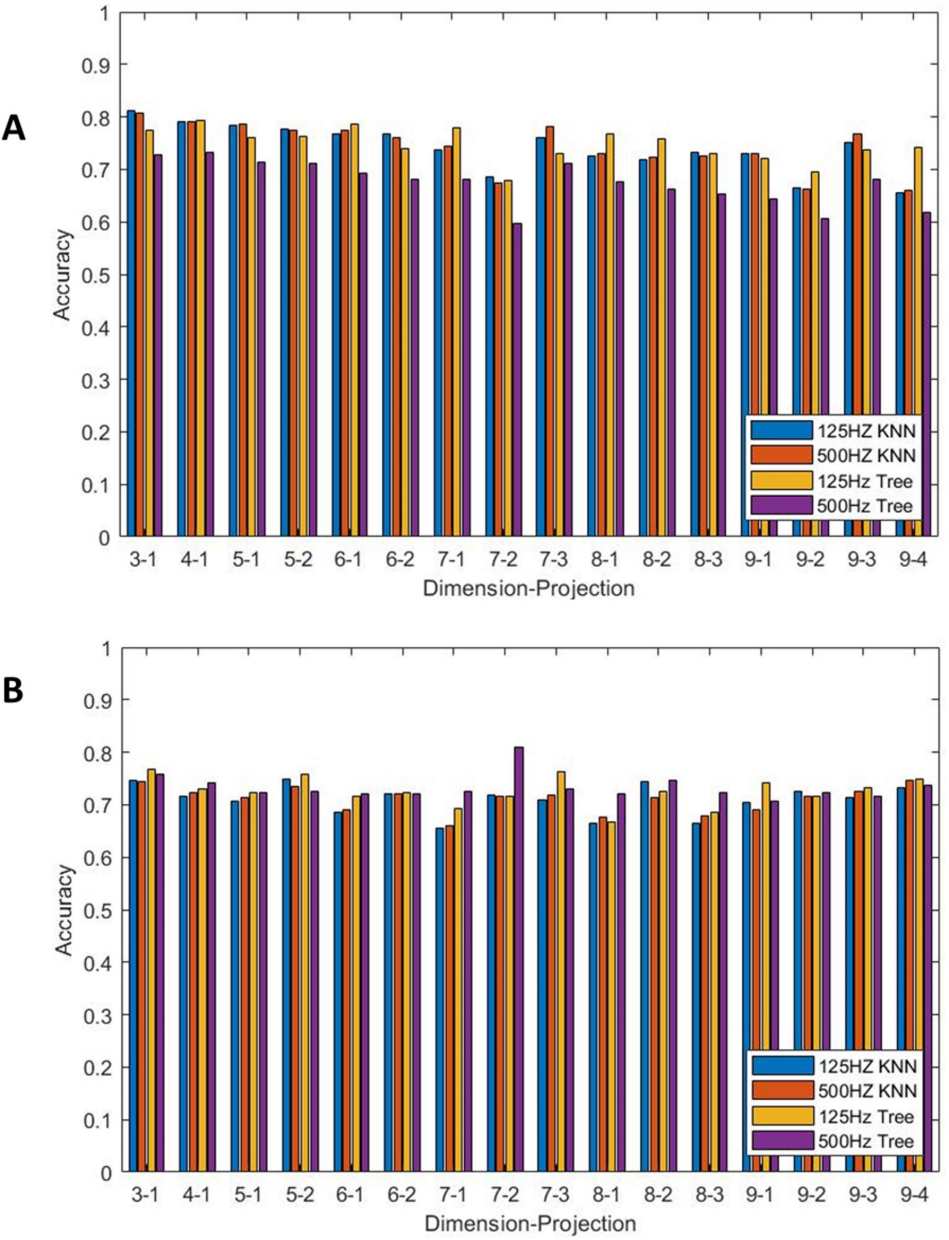
A comparison of the maximum accuracy across all 12 leads for sampling frequencies 125 Hz and 500 Hz using both KNN and decision tree models across dimensions 3–9 for all projections for (**A**) θ density features, (**B**) r density features.

Figure 2A and B show the accuracies obtained using θ and r density features respectively across dimensions 3–9 with all projections where the maximum value for the 12 leads was again taken. Again, the predominant leads for r density features were leads V3, V4 and V6, and for θ density features were leads I, aVL and V6. The accuracies were found for 125 Hz and 500 Hz sampling frequency with KNN and decision tree models.

Maximum accuracy allows for a review of the results excluding any poor performing outliers which will affect the mean. The maximum accuracy for the θ density features is 81.2% (125 Hz sampling frequency, KNN model, *N* = 3, k = 1). The general trend of the results shows that for a KNN model, 125 Hz sampling frequency only outperforms 500 Hz sampling frequency in 6 of the 16 cases with 2 cases being tied. There are 6 cases in which decision tree models with 125 Hz signals outperforms both KNN accuracies. 125 Hz signals outperform 500 Hz signals for decision tree models in all cases.

There was no obvious trend to the r density features results with most achieving around 76% accuracy. The only exception to this was a large spike for a decision tree model with 500 Hz signals with *N* = 7, k = 2 which achieved 81% accuracy. The accuracy of all 4 combinations of model and sampling frequency was generally similar with the range of accuracies for each N and k being around 2%, with the exception of *N* = 6 k = 1, *N* = 7 k = 1,2,3, *N* = 8 k = 1,3 and *N* = 9 k = 1. Decision trees with 500 Hz signals outperformed decision trees with 125 Hz signals in 8 of the 16 cases with 1 case being a tie. However, KNN only achieved the highest accuracy in 2 cases, namely *N* = 6 k = 2 and *N* = 9 k = 2, in which case it was tied with decision trees using 500 Hz signals.

**Fig. 3:**
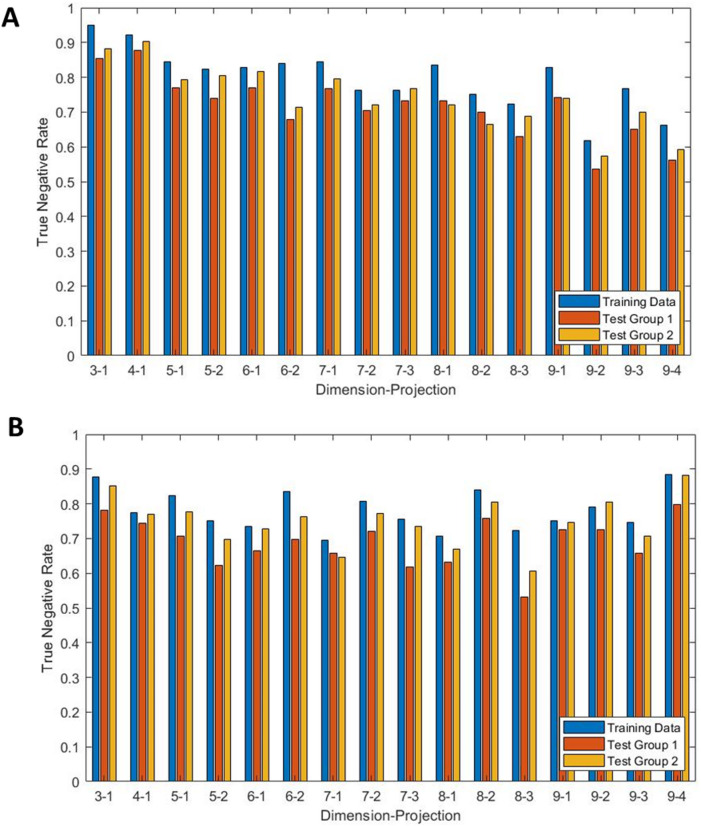
Results comparing the true negative rate for the training data and the two test sets for signals sampled at 125 Hz, a KNN model, dimensions 3–9 and all projections. Panel (**A**) shows results for θ density features and panel (**B**) for R density features.

Figure 3A, B show the comparison between the true negative rate (TNR) for the training set (125 Hz sampling frequency, KNN model with optimal parameters determined from the Bayesian optimisation) and the two Test sets described in Sect. 3.4 for *N* = 3 to 9 and all associated values of k. Panel A gives results for the θ density features. The training data achieves a maximum TNR of 95.0% for *N* = 3, k = 1 and Test groups 1 and 2 both achieve maximum TNR of 87.7% and 90.3% respectively for *N* = 4, k = 1. Panel B gives results for the R density features. The training data maximum TNR of 88.4% occurs for *N* = 9 k = 4 and the test data maximum accuracies of 79.9% and 88.3% respectively are also found for *N* = 9 k = 4.

The best results were obtained for 125 Hz sampling frequency, KNN model, *N* = 3, k = 1, and so this combination was used for the remainder of the results. Table 1 provides a breakdown of accuracies for different age bands and sexes of the patients. It was decided that all age bands under 50 would be combined rather than giving the full breakdown as in Table 5 due to the small number of patients in some bands which could give misleading results.

**Table 1:** A breakdown of accuracy by age group and sex when using 125 Hz sampling frequency, KNN model, θ density features, *N* = 3 k = 1.

Age group	Male patients (number of patients)	Female patients (number of patients)	Male and female patients (number of patients)
Under 50	70% (20)	60% (10)	66.6% (30)
50–59	82.7% (52)	77.3% (22)	81.1% (74)
60–69	88.5% (78)	83.3% (12)	87.8% (90)
70–79	84.8% (46)	77.5% (40)	81.4% (86)
80–89	83.3% (24)	77.6% (58)	79.3% (82)
All Ages	84.1% (220)	76.8% (142)	81.2% (362)

**Table 5:** A breakdown of the age groups within the dataset split by PAF and control including the number of males and females within each dataset.

Age group	Total patients	Male controls	Female controls	Male PAF	Female PAF
20–29	4	1	1	1	1
30–39	12	3	3	3	3
40–49	14	6	1	6	1
50–59	74	26	11	26	11
60–69	90	39	6	39	6
70–79	86	23	20	23	20
80–89	82	12	29	12	29
Total	362	110	71	110	71

Table 2 gives the confusion matrix for this case to highlight the difference in detecting cases and controls. In this table (and in Tables 3 and 4) the true classification was taken from the PTB-XL database labels whilst the predicted classifications came from the model output. Classification for men had a higher accuracy compared to women in all cases with a range of 5–10% difference in accuracy. Tables 3 and 4 give the confusion matrices for males and females respectively in the age band 70–79. It is clear from these tables that the main contributing factor to the change in accuracy is the model’s inability to accurately predict female PAF patients. Age band had a smaller impact with most results being within 2% of one another with the exceptions of under 50 which performed worse and 60–69 which performed better. Lyle et al.^18^ showed that the accuracy of classification of sex from the ECG declined as age increased, which suggests that male and female ECGs are more distinct for younger people which may explain the poorer performance of PAF classification for the under 50s.

**Table 2:** A confusion matrix when using 125 Hz sampling frequency, KNN model, θ density features, *N* = 3 k = 1.

True PAF	Predicted PAF	Predicted control
	123	59
True control	9	173

**Table 3:** A confusion matrix for the male patients in the age range 70–79 from Table 1.

True PAF	Predicted PAF	Predicted control
	16	7
True control	0	23

**Table 4:** A confusion matrix for the female patients in the age range 70–79 from Table 1.

True PAF	Predicted PAF	Predicted control
	11	9
True control	0	20

Following this it was decided to investigate the impact of male only and female only subsets of the PTB-XL data and also the inclusion of sex and age as features. Due to the r density features and θ density features having maximal values of around 0.3 and 0.035 respectively the ages and sexes were normalised. Bayesian optimisation was used to find the optimal parameters for the normalisation of the age and sex features. The age range was normalised in terms of two parameters Age1 and Age2 to be from Age1 to Age1 + Age2. For sex, two parameters, Sex1 and Sex2, were optimised and corresponded to the numerical label used for male and female patients respectively. The optimal values were found to be Age1 = 0.0103, Age2 = 0.9983, Sex1 = 0.0486, and Sex2 = 0.0317. The primary finding as a result of this optimisation was that the inclusion of the age and sex features often did not worsen the accuracy of the model but did not improve it either. When using the full training dataset, the inclusion of age features or sex features (or both) resulted in an overall accuracy of 81.2% matching the results found when using only θ density features. Using male or female only datasets showed a drop in accuracy with 78.6% and 66.9% accuracy respectively but the inclusion of age features to these datasets showed no change.

**Fig. 4:**
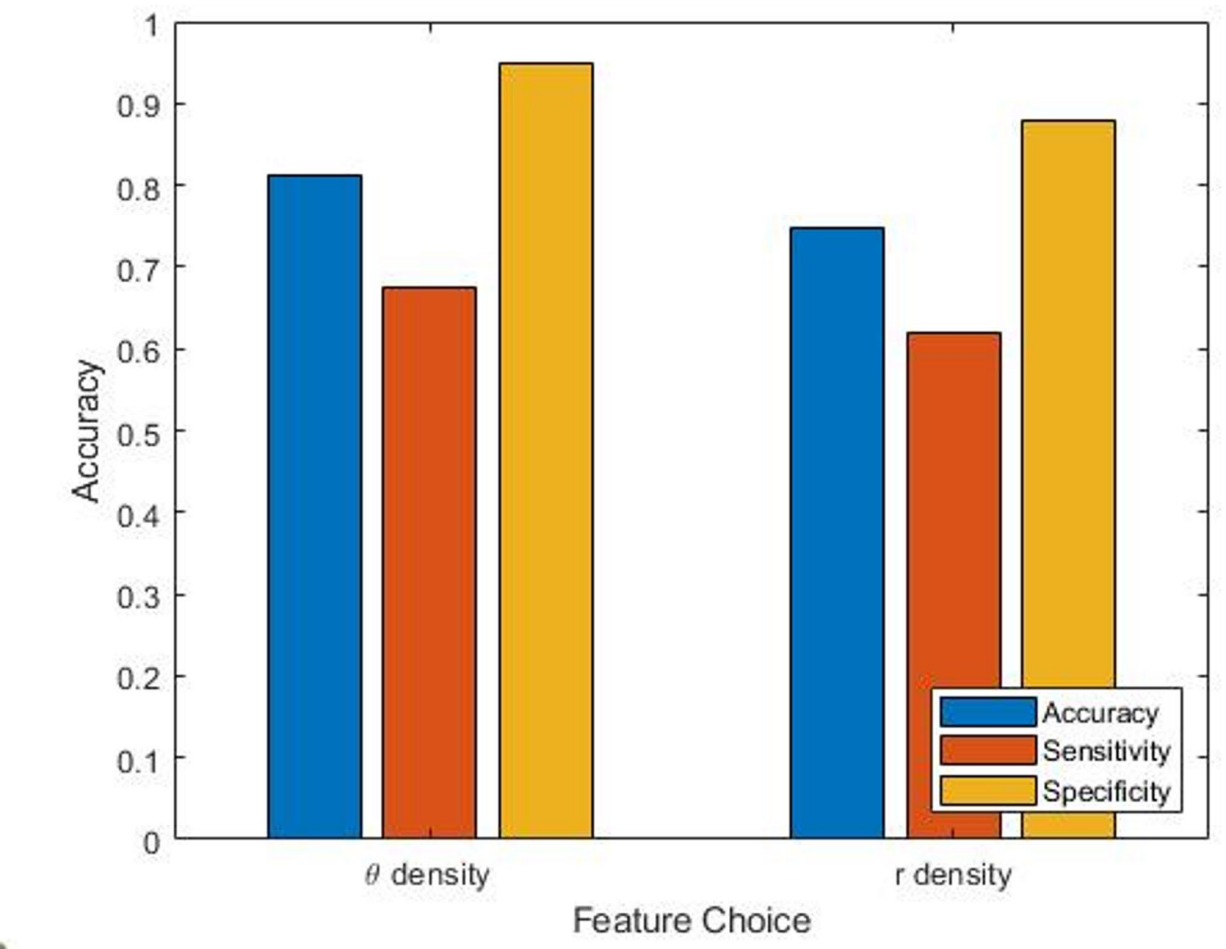
A bar chart showing the accuracy and associated sensitivity and specificity for both θ and r density for classifying PAF from sinus rhythm records. These used 125 Hz sampling frequency, KNN model and *N* = 3, k = 1.

As a result of our investigation, it was agreed that 125 Hz sampling frequency, KNN model, *N* = 3 k = 1 should be used going forward. This combination of parameters gave the highest accuracy for θ density features and a representative accuracy for r density features. Figure 4 shows the accuracy, sensitivity and specificity of each of these feature sets with these parameters. As mentioned above, θ density features achieved an accuracy of 81.2%, this is broken down into 67.4% sensitivity and 95.0% specificity. For r density features 74.9% accuracy was broken down into 61.9% sensitivity and 87.8% specificity. As a comparison point the sensitivity and specificity of decision tree models was investigated as the accuracy was similar to KNN models. It was found that the sensitivity for decision tree models was 72.5% (compared to 67.4% for KNN) and the specificity was 86.3% (compared to 95% for KNN) giving an overall accuracy of 79.4% for the best performing method (θ density features, 125 Hz sampling frequency, *N* = 4,k = 1). This shows a modest increase in sensitivity compared with the KNN model but a larger decrease in specificity, contributing to the overall decrease in accuracy. It follows that depending on the requirements for the model and whether higher sensitivity or specificity is preferred this could impact the choice of machine learning model.

Misclassification analysis was performed to investigate if there were any patients or records that were mislabelled by the algorithm in a majority of cases, which was chosen to be 75% of the classifications. Two situations were considered, namely (i) using the optimised parameters and taking the classifications from all values of N and k (16 cases), and (ii) all leads using the optimal parameters for *N* = 3, k = 1 (12 cases). When considering all values of N and k there were 21 records misclassified consistently (at least 12 of the 16 times) of which only one was a false positive (patient 7909), that is to say a control classified as a case. When looking at all leads for *N* = 3, k = 1 there were 33 misclassifications of which two were false positives (patients 7909 and 5238). Whilst PAF patients are known PAF due to the existence of a record containing an AF episode, control records are simply those without an AFIB record and so there is the possibility that they have undiagnosed PAF. The chances of this are low but in a dataset of 300 patients it is not unreasonable to expect that one or two patients could have undiagnosed PAF, and we speculate that these misclassified control patients could fall into this category. Unfortunately, there is no follow up for patients so we do not know if they were later diagnosed with PAF.

Reviewing the comorbidities for each set of misclassifications showed that a majority of misclassified records had no comorbidities; however, for all N and k those that did had only experienced conduction disturbance and myocardial infarction. Similarly for all leads with *N* = 3, k = 1 the few comorbidities were hypertrophy and ST/T change. We also reviewed the timing of the nearest AF record to the misclassified sinus rhythm records in case a pattern existed between time of recording and classification. Approximately half the records had AF records only recorded after the sinus rhythm record which raised questions as to whether records without a prior AF record should be included. A subset of the PTB-XL dataset was created matching this criterion, lowering the number of records to 238; however, when this was tested using the same optimised parameters the accuracy dropped to 75.6%. This could be as a result of using a smaller dataset, as the percentage change for each misclassified record is bigger than in a larger dataset so each misclassification penalises the accuracy of the model more. It is clear that timing of recording to the known AF episode is important but further research is needed to fully understand the impact.

## Discussion

**Fig. 5:**
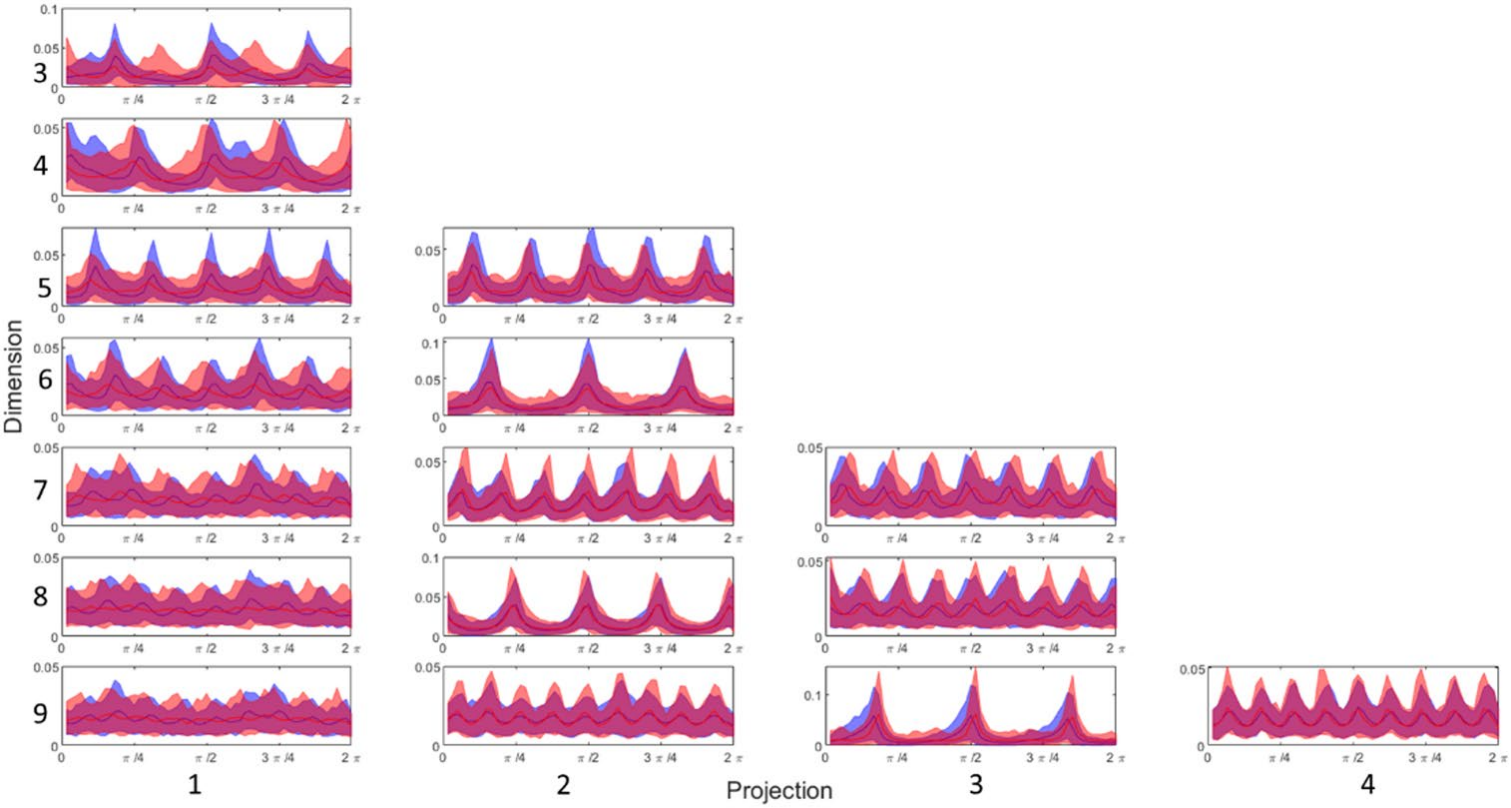
Theta density plots for lead V6, all projections for dimensions 3–9. 5th and 95th percentile intervals with PAF patients in orange and control patients in blue.

Whilst optimisation was performed on the resolution of the r and θ densities, Fig. 1 shows that they make minimal difference. This was done as a precautionary measure to ensure that the models used were not being limited by the methodology. However, it also provides further information on the changes between sinus rhythm ECGs for PAF and control patients. If the change in resolution played a significant role in the accuracy of the model it would indicate that detection of changes in the morphology of the r and θ densities would impact the overall accuracy. As this is not the case it likely instead implies that the morphological change in the signal creates a fundamental change in the shape of the attractor, whether that is a rotation of the arms or an increase or decrease in the size of the core. Figure 5 reinforces this theory with the substantial changes between PAF and control patients coming from a change in amplitude, width, or rotation of the arms (corresponding to height, width and translations of the peaks on the graph). One of the biggest changes in this plot is in the number of peaks for *N* = 3, k = 1. The doubling of the number of peaks for PAF patients could be associated with an increase in the number of arms or a change within the core of the attractor. As this is quite an obvious change to the θ density plot it would often be picked up by machine learning which explains the higher accuracy seen in the results for *N* = 3, k = 1.

Whilst the highest accuracies are achieved for θ density features derived from 125 Hz signals using a KNN model, as mentioned above, we can see a consistent trend that a sampling frequency of 125 Hz combined with a decision tree model often performs to a similar standard with both r and θ density features.

With r density features and a decision tree model, results with a sampling frequency of 500 Hz are commonly better than for 125 Hz sampling frequency as mentioned above. However, we can see that, with the exception of *N* = 7, k = 2, some of the highest accuracies come from 125 Hz signals and the differences between sampling frequencies of 125 Hz and 500 Hz is often small.

A similar review for the θ density features showed that a decision tree model using signals sampled at 125 Hz regularly achieved a similar accuracy to a KNN model for both sampling frequencies. Therefore, use of decision tree models with 125 Hz signals would provide high accuracy for both r and θ density features without compromise for certain dimension and projection combinations.

It was expected that at least one of bin density and sampling frequency would have a larger impact on the accuracy of the models than was actually seen, which raises further questions as to why not much variation was observed. Due to the nature of the SPAR method, a large proportion of the information from the original ECG is preserved which could account for the lack of impact from sampling frequency. 125 Hz is still a high enough sampling frequency to preserve all the features of the ECG and the conversion to a density plot through the SPAR method could eliminate the impact of sampling frequency changes.

The accuracy for signals sampled at 125 Hz was consistently better than for signals sampled at 500 Hz when using θ density features and a decision tree model but this difference was not seen for r density features and a decision tree model or either set of features with a KNN model. It is not clear why the combination of θ density features derived from 500 Hz signals with a decision tree model performs so poorly. This was a large contributing factor in the selection of 125 Hz for future analysis. A consideration was made to investigate lower sampling frequencies to determine if this trend continued, however the next logical step would be to decrease to 62.5 Hz. As most noise in ECG signals exists at around the 50 Hz range we felt that 62.5 Hz would be too close to this and produce erroneous results. Another consideration was to investigate sampling frequencies nearby to 125 Hz such as 80–100 Hz. As mentioned previously, 125 Hz and the other sampling frequencies were selected for their ability to be directly achieved from the original data without the need for interpolation. As the data was open source we did not have a clear understanding of the recording conditions and quality of the data and as such felt it would be unwise to attempt to interpolate at other sampling frequencies. We suggest that future studies may wish to investigate the impact of this sampling frequency further using the analysis methods discussed in this study.

We note that accuracy of prediction for males is higher than for females. This may be due to the fact that our dataset is imbalanced in regard to sex as it consists of 61% males ECGs and 39% female ECGs. Future work could consider whether a more balanced dataset gives more balanced results with regard to sex.

Figure 3 gives an insight into the comparison between the sensitivity and specificity of the model. Comparison of the results of Test group1 and Test group 2 shows that the presence of comorbidities lowers the TNR of the model due to the consistent outperformance of Test group 2 over Test group 1. As the training dataset is balanced between cases and controls the accuracy for the training data is the mean of the specificity and sensitivity. Now knowing the TNR and comparing with the accuracy from Fig. 2, it follows that the sensitivity is far lower than the specificity (this can also be seen in Table 2).

The impact of comorbidities on the model’s accuracy could help explain this as the PAF patients were selected irrespective of if comorbidities were present or not. This is reinforced by the work with misclassifications which showed that certain comorbidities consistently appeared in records misclassified at least 75% of the time. In future, further investigation into the impact of comorbidities may give significant increases in sensitivity, however this may require collection of a new dataset with enough patients to investigate each comorbidity without overfitting. Alternatively, the timing of the PAF episodes could play an important role in the overall accuracy of the model. In Attia et al.^15^ all PAF episodes were within 31 days of the chosen records. However, with our data the time between the episode and record is inconsistent. In some cases, the sinus rhythm record is prior to the known PAF episode but due to the low likelihood that this was the patient’s first episode these records were still included. Future work to evaluate the impact of time between episode and recording could help with detection and impact the time before an ECG is taken in primary care.

The comparison between the training data and Test group 2 gives a good representation of the ability for the model to be applied to other data sets. The training data only included control patients without comorbidities and so the drop in TNR between the training data and Test set 1, similarly with Test set 2 and Test set 1, reinforces the negative impact of comorbidities within the data. It should be noted that the original data set contained 44 diagnostic statements which were subsequently collapsed to 5 superclasses which were used within the dataset. Due to the size of the dataset it is also likely that each comorbidity has very few occurrences which would cause difficulty for the model to distinguish these changes. The TNR for Test set 2 is often similar to the training data, particularly when using r density features. The difference between these TNRs likely comes from a small amount of overfitting with the training data which could stem from the size of the data set. Test set 2 is 18 times larger than the training data which would therefore amplify the effects of overfitting.

## Conclusions

As a result of this study, our principal findings are as follows:

- The resolution of both the r and θ densities has a minimal impact on the overall accuracy of the models.
- θ density features often give the best results when using KNN models whereas r density features generally give the best results with decision tree models. However, in many cases the results are similar.
- The sampling frequency of the signals has the most impact when using a decision tree model with θ density features but otherwise does not cause a large change in accuracy.
- *N* = 3, k = 1 and *N* = 4, k = 1 consistently achieved the highest accuracies for θ density features, KNN models, irrespective of sampling frequency. This trend was less obvious for both r density features and decision tree models although the accuracies were usually more consistent across the choice of N and k in this case. For this reason, *N* = 3 k = 1 was taken as the choice of dimension and projection to achieve the best accuracy.
- This study demonstrated a maximum accuracy of 81.2% when using a KNN model. However the best sensitivity of 72.5% was achieved using a decision tree model. Studies investigating the current best practice of long term monitoring achieved a sensitivity of 34% with 30 day monitoring. It should be noted that this metric shows that 34% of subjects developed AF in the 30 day period but does not consider those who may have developed AF afterwards. Nonetheless, our method requires only a 10 s recording in sinus rhythm to achieve a result with twice the sensitivity showing a clear improvement on time and a potentially double increase in sensitivity.

## Materials and methods

### Dataset choice

PAF has a higher prevalence in older patients^22^ and so creating a balanced dataset required a database with a large number of control patients to allow for suitable matching with PAF patients. We felt it was important for control patients to match both age and sex for this reason and so PTB-XL^23–25^ was chosen which contains 21,799 10 s, 12 lead ECG signals from 18,869 patients experiencing a range of cardiac conditions confirmed by cardiologists. All ECGs were sampled at 500 Hz and meta data included age, sex and comorbidities.

The PTB-XL dataset supplementary paper^23^ provides information on the rhythm and diagnostic changes incorporated as part of the dataset. The rhythm statements include AFIB (atrial fibrillation) and SR (sinus rhythm) allowing for a determination of PAF patients for whom the sinus rhythm records were selected. The type of AF was not included as part of the database so there is a potential for persistent AF patients to be included.

### Dataset breakdown

**Fig. 6:**
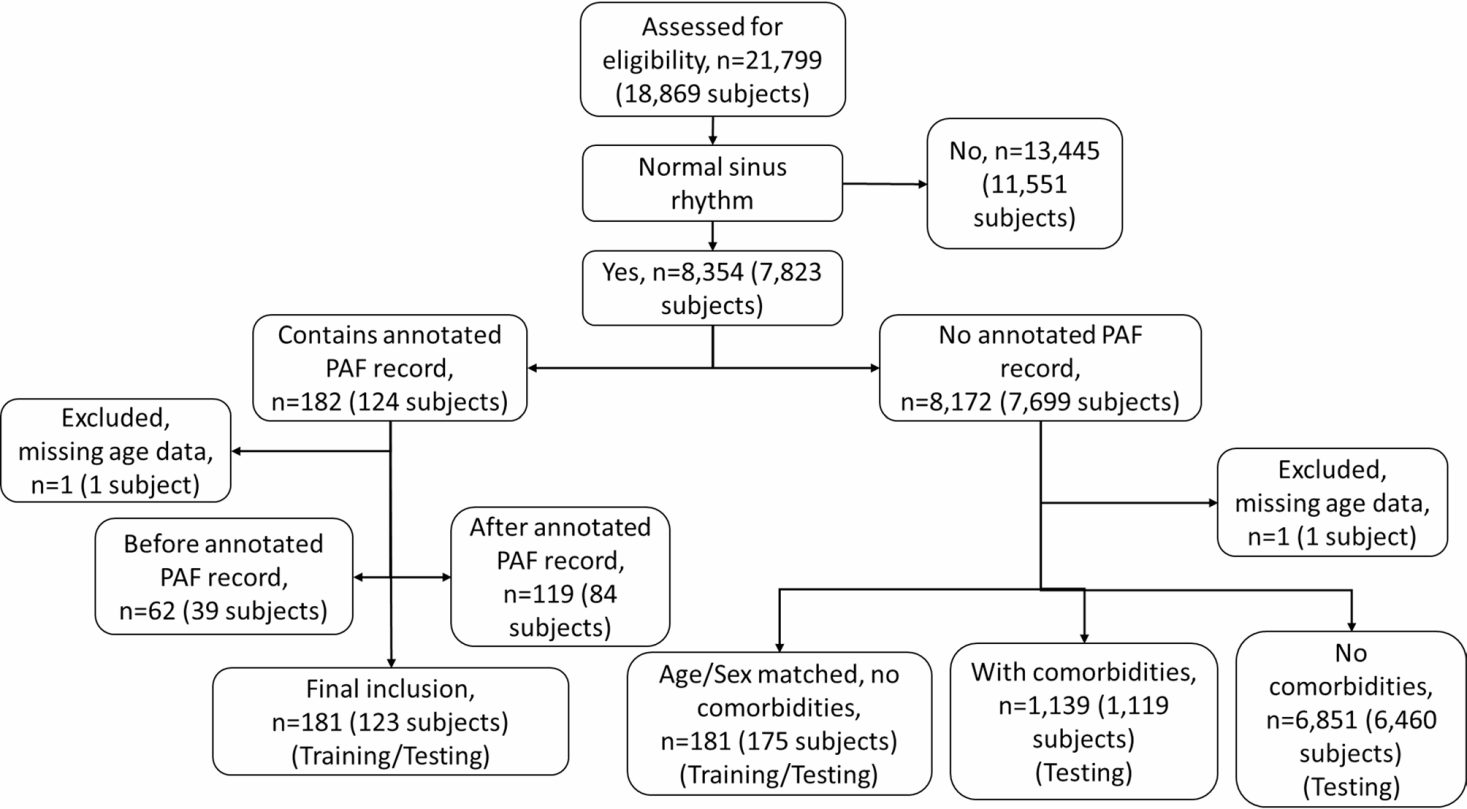
A CONSORT diagram outlining the process of selecting the records for training and testing from the PTB-XL database.

All 123 patients with AF and sinus rhythm records were chosen and all sinus rhythm records from these patients were used to maximise the available data. Figure 6 shows a CONSORT diagram of the dataset and how it was constructed from the full PTB-XL database. It is unlikely that the AF recordings taken as part of this study were the first AF episodes experienced by the patients and so it was expected that all sinus rhythm records were taken after an AF episode; however not all may be confirmed within PTB-XL. Further investigation showed that any sinus rhythm records taken before an AF episode were at most 1 month prior.

A balanced dataset was then created by selecting control patients who had no AF records or comorbidities but did have a sinus rhythm record. To account for the increased prevalence of PAF in older patients and the disparity between men and women the control records were matched by age (within a decade) and sex to the case records.

This resulted in 362 records, split equally into 181 cases and 181 controls across 298 unique patients. Table 5 shows the distribution of these records across age groups. After matching, this dataset is skewed more towards male records with 220 male records to 142 female records. PAF is more prevalent in men but the life expectancy of women is longer so there is a similar number of men and women overall with PAF^26,27^. However, in the PTB-XL database, there is a higher number of male patients overall, but the highest age ranges contain a larger number of female patients.

### SPAR method

**Fig. 7:**
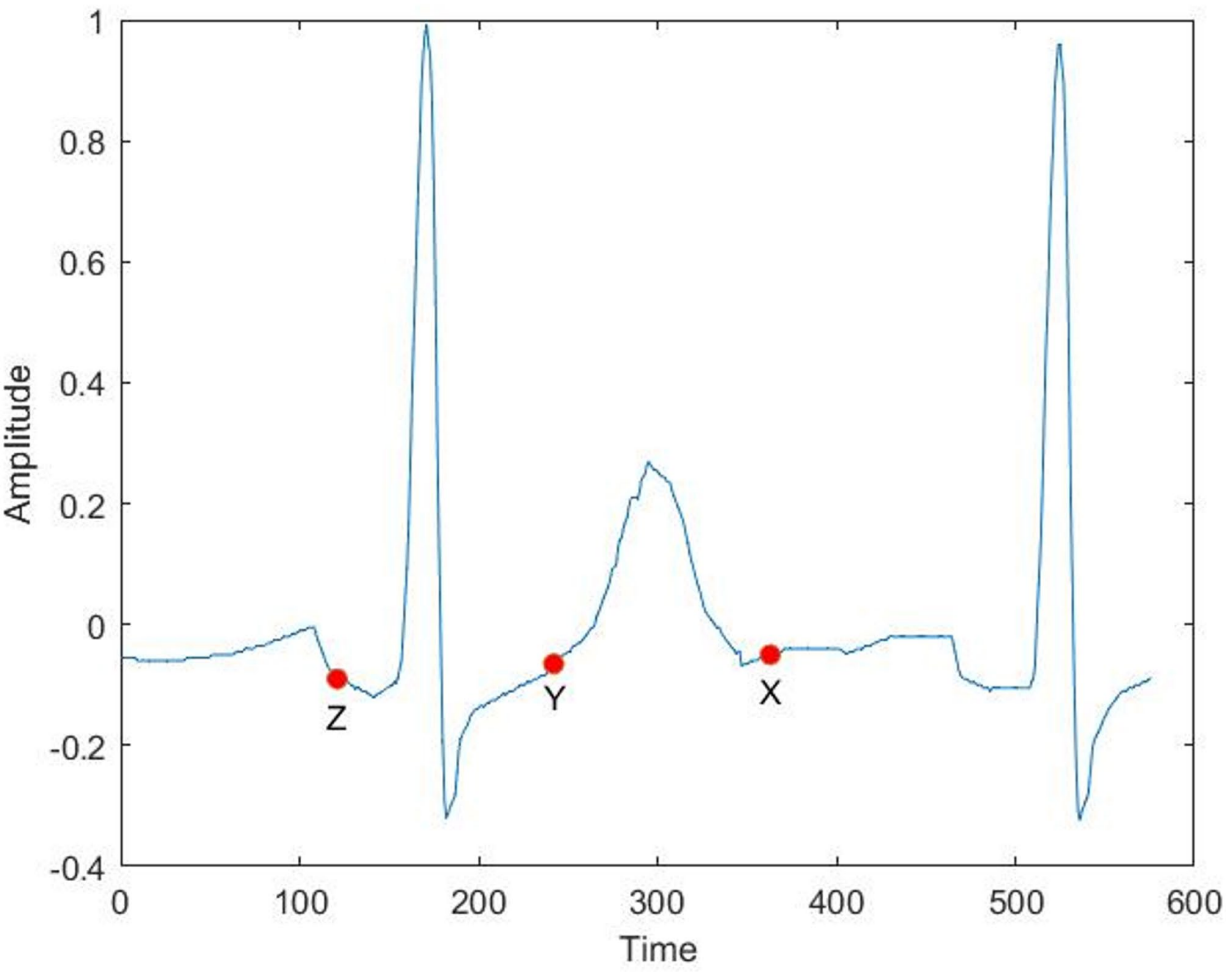
A visual representation of Takens’ delay coordinates.

**Fig. 8:**
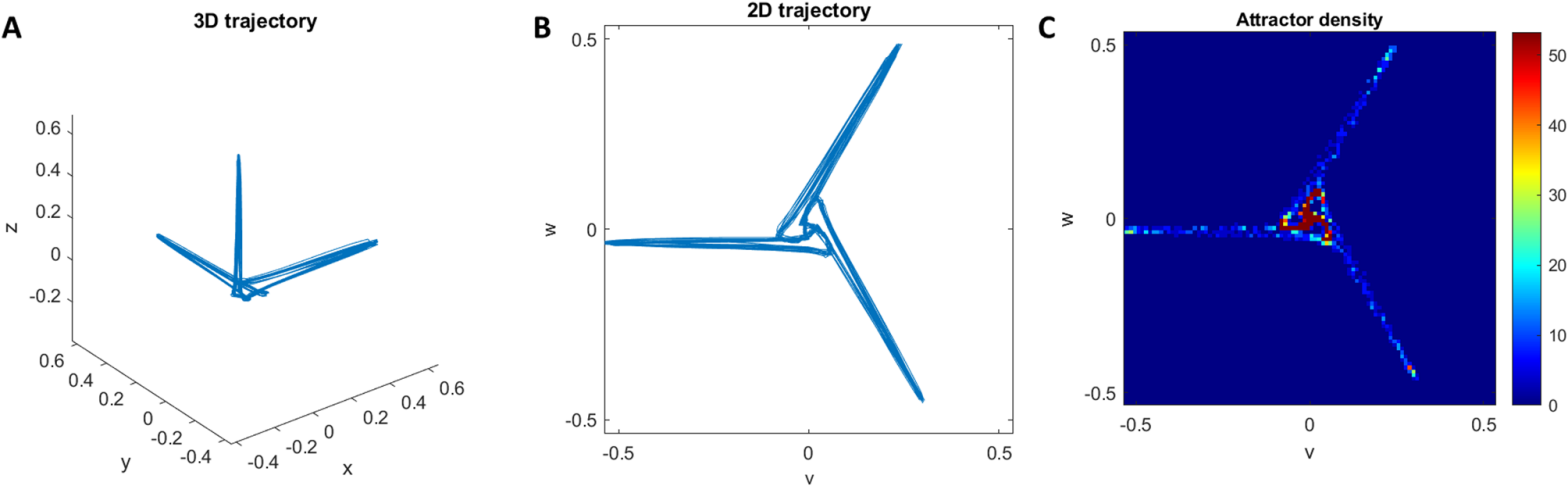
(**A**) Takens’ delay coordinates plotted in 3 dimensions. (**B**) The projection into 2 dimensions to reduce the impact of baseline wander. (**C**) The conversion into a density plot to eliminate loss of information through overlapping lines.

Attractors have become commonplace as a method to understand the behaviour of dynamical systems defined by a system of ordinary differential equations (ODEs). The method involves plotting a trajectory in phase space parameterised by time where each of the variables corresponds to one axis. When working with time series data from a single signal, such a plot is not possible as there is only one variable. Takens solved this problem by defining several delay coordinates from a single signal which are separated by a fixed time delay as shown in Fig. 7^28^. The Symmetric Projection Attractor Reconstruction method^16,17,29^ generates a two-dimensional image from an approximately periodic signal, such as an ECG, using Takens’ delay coordinates. The full SPAR method is as follows. The average cycle length of an ECG is calculated by averaging the distance between successive R peaks. Three equally spaced points are then placed on the signal, a distance of one third of the average cycle length apart (see Fig. 7). As with the attractor construction mentioned above, each of these three points is plotted on a different axis in 3D space as the points traverse the signal. Once all the signal has been traversed, we are left with a 3D reconstructed attractor of the signal (see Fig. 8A). However, it still includes the most variable part of the signal, baseline wander. By viewing the attractor in the direction of the vector (1,1,1) the effect of baseline wander is significantly reduced^17^ (see Fig. 8B). This 2D projection is converted to a density plot to highlight the overlapping of lines and avoid any loss of information (see Fig. 8C). Any change in the morphology of the signal will then result in a change in the shape of the attractor.

The SPAR method was generalised to use a higher number of delay coordinates^30^ but always resulting in a 2D attractor. However, taking 5 or more points results in several 2D attractors that can be generated. The number of delay coordinates and choice of 2D representation are known as the dimension (N) and projection (k) respectively.

**Fig. 9:**
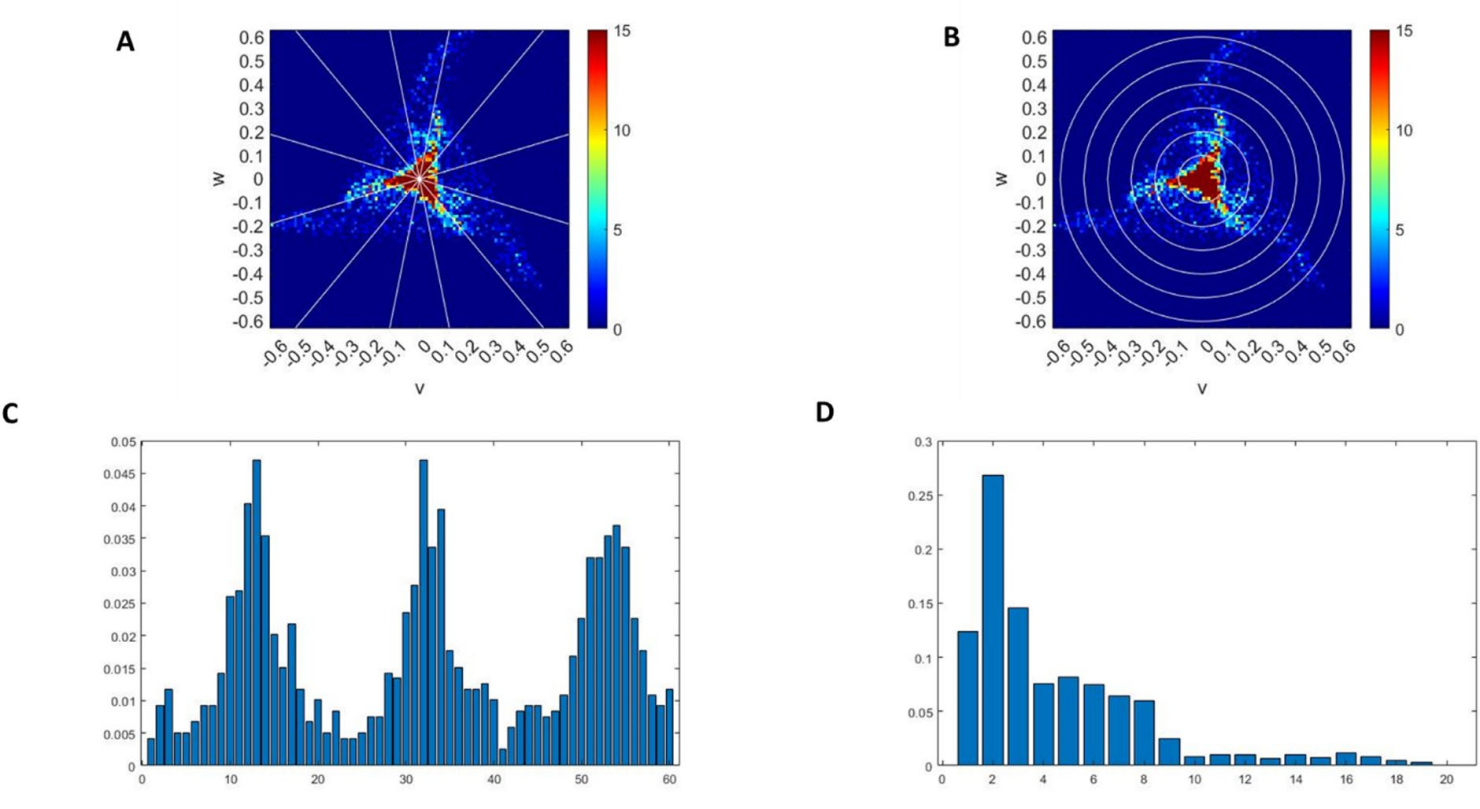
Panels (**A**) and (**B**) give a visual representation of the theta density wedges and r density annuli respectively. Panels (**C**) and (**D**) give the plot of standard attractor densities corresponding to panels (**A**) and (**B**).

Previous work has used attractor images as the input for deep learning^31^ but for machine learning numeric features need to be extracted for use as inputs. Two main sets of features that were used for this study are generated from the attractor’s r density and θ density. The r density measures the density between two annuli of increasing distance from the origin (see Fig. 9B). This provides a good indication of the size and relative density of the core of the attractor. The θ density provides the density of wedges taken between lines stemming from the origin of the attractor (see Fig. 9A). These give an indication of the density and position of the arms of the attractor indicating any rotation. Plotting the density against θ and r (see Fig. 9C and D respectively) allows for mathematical comparisons between attractors, rather than needing a technique such as deep learning for comparison of images. The resolution, often referred to as the number of bins, can also be varied as part of our machine learning. For the r density this corresponds the number of annuli taken on the attractor. For the θ density this corresponds to the number of wedges around the attractor.

### Resampling and optimisation

The PTB-XL data is provided at 500 Hz. In order to evaluate the impact of sampling frequency a second feature set resampled to 125 Hz was created. Performing all analysis on both feature sets gave a clear indication of how sampling frequency changed the classification accuracy. 125 Hz was specifically chosen for ease of resampling and for its commonality in clinical ECG machines^32^. 125 Hz is one quarter of the frequency of 500 Hz which allows for resampling without interpolation of data and has also been shown to provide good results with heart rate variability analysis^33^.

The number of bins for the r and θ densities was optimised by taking different values and the results were compared. The number of bins for the r density was taken as 10, 20, 30, 40, 50, 60, 70, 150, 300 and for the θ density was taken as 20, 40, 60, 80, 100, 120, 250, 500.

The machine learning models used were created using K nearest neighbours (KNN) and decision tree algorithms. Each model was provided with the r and θ densities for each of the 12 leads across all combinations of dimension, ranging from 3 to 9, and all associated projections separately.

Machine learning tools are often very sensitive to data they have previously seen. To avoid inflating the performance of the models a leave-one-patient-out cross validation was used. All records from a single patient were removed and the remainder of the feature table was used as the training data. The classification for each record of the excluded patient was then obtained and saved. This process was repeated for each patient and the overall accuracy was determined by the percentage of correct record classifications.

Bayesian optimisation was used to optimise the hyperparameters to determine the combination which gave the highest cross validation accuracy for each model. Table 6 summarises the parameters used for this optimisation and the corresponding range of values taken for each parameter.

**Table 6:** A summary of the parameters and corresponding ranges taken for machine learning optimisation.

Machine learning method	Parameter	Values
KNN	distance metric	10 categorical variables (cityblock, chebychev, correlation, cosine, Euclidean, hamming, jaccard, minkowski, seuclidean, spearman)
	Number of neighbours	Odd values from 1 to 99
Decision tree	Minimum leaf size	1 to 182

From these results the optimal parameters for each lead, dimension and projection combination and for a range of bin values were obtained. In many cases, the highest accuracy was obtained for one set of values of the hyperparameters. However, when there were several sets of values corresponding to the highest accuracy, the mode across lead, dimension, and projection was taken. It was then checked that this combination of parameters was one of the optimal options. If not, the first entry in the list was taken.

All of the available PAF records from PTB-XL were used in our dataset, as described above. However, there were many control records that were not used. These were used to form two further datasets consisting of comorbidity controls and healthy controls (known as Test group 1 and Test group 2 respectively). Test group 1 contained 1139 records from 1119 patients and Test group 2 contained 6851 records from 6460 patients. A machine learning model was then trained using all of the training data and the optimal hyperparameters and this was used to classify each of the records in these two additional test datasets. For these datasets, we report the true negative rate and compare this with the true negative rate obtained from the cross validation.

## Data Availability

The datasets analysed for this study can be found on the Physionet website (https:/physionet.org/content/ptb-xl/1.0.3) .
